# Accuracy of whole-genome sequence imputation using hybrid peeling in large pedigreed livestock populations

**DOI:** 10.1186/s12711-020-00536-8

**Published:** 2020-04-06

**Authors:** Roger Ros-Freixedes, Andrew Whalen, Ching-Yi Chen, Gregor Gorjanc, William O. Herring, Alan J. Mileham, John M. Hickey

**Affiliations:** 1 The Roslin Institute and Royal (Dick) School of Veterinary Studies, The University of Edinburgh, Easter Bush, Midlothian, Scotland, UK; 2 Departament de Ciència Animal, Universitat de Lleida-Agrotecnio Center, Lleida, Spain; 3 The Pig Improvement Company, Genus plc, 100 Bluegrass Commons Blvd Ste 2200, Hendersonville, TN 37075 USA; 4 Genus plc, 1525 River Road, Deforest, WI 53532 USA

## Abstract

**Background:**

The coupling of appropriate sequencing strategies and imputation methods is critical for assembling large whole-genome sequence datasets from livestock populations for research and breeding. In this paper, we describe and validate the coupling of a sequencing strategy with the imputation method hybrid peeling in real animal breeding settings.

**Methods:**

We used data from four pig populations of different size (18,349 to 107,815 individuals) that were widely genotyped at densities between 15,000 and 75,000 markers genome-wide. Around 2% of the individuals in each population were sequenced (most of them at 1× or 2× and 37–92 individuals per population, totalling 284, at 15–30×). We imputed whole-genome sequence data with hybrid peeling. We evaluated the imputation accuracy by removing the sequence data of the 284 individuals with high coverage, using a leave-one-out design. We simulated data that mimicked the sequencing strategy used in the real populations to quantify the factors that affected the individual-wise and variant-wise imputation accuracies using regression trees.

**Results:**

Imputation accuracy was high for the majority of individuals in all four populations (median individual-wise dosage correlation: 0.97). Imputation accuracy was lower for individuals in the earliest generations of each population than for the rest, due to the lack of marker array data for themselves and their ancestors. The main factors that determined the individual-wise imputation accuracy were the genotyping status, the availability of marker array data for immediate ancestors, and the degree of connectedness to the rest of the population, but sequencing coverage of the relatives had no effect. The main factors that determined variant-wise imputation accuracy were the minor allele frequency and the number of individuals with sequencing coverage at each variant site. Results were validated with the empirical observations.

**Conclusions:**

We demonstrate that the coupling of an appropriate sequencing strategy and hybrid peeling is a powerful strategy for generating whole-genome sequence data with high accuracy in large pedigreed populations where only a small fraction of individuals (2%) had been sequenced, mostly at low coverage. This is a critical step for the successful implementation of whole-genome sequence data for genomic prediction and fine-mapping of causal variants.

## Background

Sequence data has the potential to empower the identification of causal variants that underlie quantitative traits or diseases [1–4], enhance livestock breeding [5–7], and increase the precision and scope of population genetic studies [8, 9]. For sequence data to be used routinely in research and breeding, low-cost sequencing strategies must be deployed in order to assemble large datasets that capture most of the sequence diversity in a population and enable harnessing of its potential. One possible strategy is to sequence a subset of the individuals in a population at low coverage and then to perform imputation of whole-genome sequence data for the remaining individuals [10–12].

Such a strategy is likely to perform well in livestock breeding populations, in which individuals have a high degree of relatedness, allowing low-coverage sequence data to be pooled across individuals that share a haplotype and imputed to individuals who share that haplotype. Due to the implementation of genomic selection in livestock breeding populations, many individuals in breeding nucleus populations have already been genotyped with marker arrays. This genotype data can be used to identify the individuals that share haplotype segments and to select individuals for sequencing that will be more informative from an imputation perspective given a limited budget [13, 14].

We have recently proposed ‘hybrid peeling’ [15], a fast and accurate imputation method explicitly designed for jointly calling, phasing and imputing whole-genome sequence data in large and complex multi-generational pedigreed populations in which individuals can be sequenced at variable coverage or not sequenced at all. Hybrid peeling is a two-step process. In the first step, multi-locus iterative peeling is performed to estimate the segregation probabilities for a subset of segregating sites (e.g., the markers on a genotyping array). In the second step, the segregation probabilities are used to perform fast single-locus iterative peeling on every segregating site discovered in the genome. This two-step process allows the computationally demanding multi-locus peeling step to be performed on only a subset of the variants, while still leveraging linkage information for the remaining variants.

These properties make hybrid peeling a very appealing imputation method for the cost-effective generation of whole-genome sequence data for large pedigreed populations that have already been extensively genotyped using marker arrays and in which a small proportion of the individuals have been sequenced with variable coverage. In the situations described, the sequence data will be sparsely distributed across the pedigree and there may be great variability in the amount of data to which each individual is exposed. Understanding which factors affect individual-wise and variant-wise imputation accuracy and how their effects are mediated is important for determining how this sequencing strategy, together with hybrid peeling, performs in real settings that are common in animal breeding and for enabling accuracy-aware quality control of the imputed data before downstream analyses. Such knowledge could be used in the future to design cost-effective routine whole-genome sequencing strategies.

The objectives of this study were to: (i) demonstrate whether whole-genome sequence data could be imputed with high accuracy in a variety of pig pedigrees when small subsets of individuals are sequenced, mostly at low coverage; (ii) quantify the factors that determine the individual-wise and variant-wise imputation accuracy; and (iii) quantify the impact of data misassignment and pedigree errors on imputation accuracy. Our results showed that high overall imputation accuracies can be achieved for whole-genome sequence data in large pedigreed populations using hybrid peeling provided that the individuals are connected to a sufficient number of informative relatives with marker array or sequence data. Our results have implications for the practical implementation of sequencing and imputation strategies.

## Methods

We structured the study in three tests. In Test 1, we evaluated the imputation accuracy of hybrid peeling in four populations of different sizes. In Test 2, we used regression trees and simulated data based on three real pedigrees to quantify which factors determined the individual-wise and variant-wise imputation accuracy of hybrid peeling. Then, we used the observations in the real data to validate the findings and to predict individuals with low imputation accuracy. In Test 3, we evaluated the potential impact that data misassignment and pedigree errors could have on the imputation accuracy. In the following sections, we first describe how the data was generated and then how the different tests were performed.

## Real data

### Populations and sequencing strategy

We performed whole-genome sequencing of 4427 individuals from four commercial pig breeding lines (Genus PIC, Hendersonville, TN) using a total coverage of approximately 18,514×. To account for a range of population sizes, the number of individuals in each population was 18,349 (20 k), 34,425 (35 k), 68,777 (70 k), or 107,815 (110 k). Approximately 2% (1.7–2.5%) of the individuals in each population were sequenced, mostly at low coverage. The number of individuals sequenced and the coverage at which they were sequenced are summarized for each population in Table 1.

**Table 1:** Distribution of sequencing coverages by population

Population	Individuals sequenced	Individuals sequenced by coverage				Total coverage
		1x	2x	5x	15–30x	
20 k	445	217	176	15	37	1852x
35 k	760	394	274	27	65	3192x
70 k	1366	685	545	44	92	5280x
110 k	1856	1044	649	73	90	8190x

We selected the individuals and the coverage at which they were sequenced using a three-step strategy: (1) first, we selected sires and dams that contributed most genotyped progeny in the pedigree (referred to as ‘top sires and dams’) to be respectively sequenced at 2× and 1×; (2) then, we used AlphaSeqOpt part 1 [13] to identify the individuals whose haplotypes represented the greatest proportion of the population haplotypes (referred to as ‘focal individuals’) and to determine an optimal level of sequencing coverage between 0× and 30× for these individuals and their immediate ancestors (i.e., parents and grandparents) under a total cost constraint; and (3) finally, we used the AlphaSeqOpt part 2 [14] to identify individuals that carried haplotypes whith a low cumulative coverage (i.e., lower than, 10×) and distributed 1× sequencing amongst those individuals so that the cumulative coverage on the haplotypes could be increased (i.e., at or above 10×). AlphaSeqOpt used haplotypes inferred from marker array genotypes (GGP-Porcine HD BeadChip; GeneSeek, Lincoln, NE), which were phased with AlphaPhase [16] and imputed with AlphaImpute [17]. The sequencing resources were split so that approximately 30% of the sequencing resources were used for sequencing the top sires at 2×, 15% for the top dams at 1×, 25% for the focal individuals and their immediate ancestors at variable coverage [13], and the remaining 30% for individuals that carried under-sequenced haplotypes at 1× [14]. In step 2, we identified 284 individuals across the four populations who were sequenced at high coverage (15× or 30×). Many of these individuals sequenced at high coverage belonged to early generations of the pedigree of each population. The rest of the sequenced individuals were sequenced at low coverage (1×, 2× or 5×).

We sorted the pedigrees of each population so that parents appeared before their progeny. Thus, relative position in the pedigree was used as a proxy for the generation to which an individual belonged.

### Sequencing and data processing

Tissue samples were collected from ear punches or tail clippings. Genomic DNA was extracted using Qiagen DNeasy 96 Blood & Tissue kits (Qiagen Ltd., Mississauga, ON, Canada). Paired-end library preparation was conducted using the TruSeq DNA PCR-free protocol (Illumina, San Diego, CA). Libraries for sequencing at low coverage (1× to 5×) were produced with an average insert size of 350 base pairs and sequenced on a HiSeq 4000 instrument (Illumina, San Diego, CA). Libraries for sequencing at high coverage (15× or 30×) were produced with an average insert size of 550 base pairs and sequenced on a HiSeq X instrument (Illumina, San Diego, CA). All libraries were sequenced at Edinburgh Genomics (Edinburgh Genomics, University of Edinburgh, Edinburgh, UK). Most pigs were also genotyped either at low density (LD; 15,000 markers) using the GGP-Porcine LD BeadChip (GeneSeek, Lincoln, NE) or at high density (HD; 75,000 markers) using the GGP-Porcine HD BeadChip (GeneSeek, Lincoln, NE).

DNA sequence reads were pre-processed using Trimmomatic [18] to remove adapter sequences from the reads. The reads were then aligned to the reference genome *Sscrofa11.1* (GenBank accession: GCA_000003025.6; [19]) using the BWA-MEM algorithm [20]. Duplicates were marked with Picard (http://broadinstitute.github.io/picard). Single nucleotide polymorphisms (SNPs) were identified with the variant caller GATK HaplotypeCaller (GATK 3.8.0; [21, 22]) using default settings. Between 20 and 30 million SNPs were discovered in each population.

To avoid biases towards the reference allele introduced by GATK when applied on low-coverage sequence data, we extracted the read counts supporting each allele at each variant site with a pile-up function using the pipeline described in [23]. This pipeline uses the tool pysam (version 0.13.0; https://github.com/pysam-developers/pysam), which is a wrapper around htslib and the samtools package [24]. We extracted the read counts for all biallelic SNP positions, after filtering out variants with a mean coverage 3 times greater than the average realized coverage (considered as indicative of potential repetitive regions) with VCFtools [25].

We performed additional quality control on the pedigree by determining the number of Mendelian inconsistencies (percentage of opposing homozygous) between each parent-progeny pair. We applied the following criteria: (1) we removed marker array or sequence data of an individual, when the genotype data was incompatible with that of all its available parents and progeny (this was done because it could indicate data misassignment for that individual); (2) we removed parent-progeny pedigree links when the genotype data available was incompatible for only a pair of individuals but not for their other parents and progeny; and (3) we created a dummy parent with no genotype data when the genotype data of a group of littermates was incompatible with one of its parents but both the parent and the littermates were not incompatible with the rest of their parents and progeny (this was done to preserve the full-sib relationship between those individuals).

## Simulated data

In order to test the factors that influenced imputation accuracy, we simulated genetic data for three populations of different sizes: 15,187 (15 k), 29,974 (30 k), and 64,598 (65 k) individuals. The pedigrees of these populations were a subset of the real pedigrees of the 20 k, 35 k, and 110 k populations used for the analyses of real data. As in the analyses of real data, the pedigrees were sorted so that parents appeared before their progeny. Genomic data for each population were simulated using the software AlphaSim [26]. Each simulation was repeated twice and results were averaged across repetitions. Below, we present only a brief description of the simulation strategy. The full details of the simulation are described in a companion paper [27].

Genomic data were simulated for 20 chromosomes, each 100 cM long. In total, 150,000 SNPs per chromosome (3 million SNPs genome-wide) were simulated in order to represent whole-genome sequence. A subset of 3000 SNPs per chromosome (60,000 SNPs genome-wide) was used as a high-density marker array (HD). A smaller subset of 300 SNPs per chromosome (6000 SNPs genome-wide) nested within the HD marker array was used as a low-density marker array (LD). Each individual was assigned HD or LD marker array data based on the density at which they were genotyped in real data. The sequence read counts for each individual and SNP were simulated by sampling sequence reads using a Poisson-gamma model that gave variable sequenceability at each SNP and variable number of reads for each individual at each SNP [10, 28].

The individuals to be sequenced and their sequencing coverage were selected using a combination of pedigree- and haplotype-based methods that mimicked the sequencing strategy that was used for the real data. The total level of investment for sequencing was equivalent to the cost of sequencing 2% of the population at 2×, and thus resulted in a similar number of sequenced individuals as in the real data.

## Imputation using hybrid peeling

Imputation was performed in each population separately using hybrid peeling, as implemented in AlphaPeel [15], with the default settings. Hybrid peeling extends the methods of Kerr and Kinghorn [29] for single-locus iterative peeling and of Meuwissen and Goddard [30] for multi-locus iterative peeling to efficiently call, phase and impute whole-genome sequence data in complex multi-generational pedigrees. Multi-locus iterative peeling was performed on all available marker array data to estimate the segregation probabilities for each individual. The individuals genotyped with LD marker arrays were not imputed to HD prior to this step. The segregation probabilities were used for segregation-aware single-locus iterative peeling for the variant sites genome-wide.

## Imputation accuracy tests

### Test 1: Imputation accuracy in populations of different size

The imputation accuracy in the real data was estimated using a leave-one-out design. In each leave-one-out round, hybrid peeling was performed after removing the sequence data of one of the 284 individuals that were sequenced at high coverage (either 15 or 30×) in the four populations. We used the genotypes imputed for these individuals using the full data as the true genotypes. To reduce computational requirements, accuracy was assessed on a random subset of 50,000 non-consecutive SNPs from chromosome 5, which included all the markers from the arrays that map to this chromosome (~ 3000). Tests in other chromosomes gave similar results.

We measured individual-wise and variant-wise imputation accuracy with the correlation between the true genotypes and imputed dosages. The dosage correlation was calculated after correcting for minor allele frequency (MAF), as recommended by Calus et al. [31]. To facilitate comparison with other studies that report the uncorrected (raw) allele dosage correlations, in the context of this study we found that MAF-corrected correlations of 0.75, 0.80, 0.85, 0.90, and 0.95 were respectively equivalent to the raw correlations of 0.89, 0.91, 0.93, 0.96, and 0.98. For the variant-wise imputation accuracy, we excluded the individuals that had the lowest imputation accuracy, predicted as described in Test 2.

### Test 2: Factors that affect individual-wise and variant-wise imputation accuracy

We assessed the factors that influenced imputation accuracy in the simulated data. We used simulated data to provide a much larger sample size where the true genotypes were known. We ran single-locus peeling on a random subset of 5000 non-consecutive SNPs taken from across three chromosomes to reduce computational requirements, although the full set of 20 chromosomes were simulated to represent realistic genetic architecture and haplotype diversity for the haplotype-based method AlphaSeqOpt. We assessed the factors that influenced imputation accuracy by building regression trees. The regression trees were built using the data from 219,518 simulated individuals and 30,000 variants (5000 variants from each population and replicate). The regression trees were built using the ‘rpart’ R package [32], allowing partitions that increased the R^2^ of the model by 0.005 at each step. Consecutive binary partitions based on the same variable were considered as multi-part.

The regression tree for the individual-wise imputation accuracy was based on the amount of information that was available for the individual itself and its close relatives (4 relationship levels: grandparents, parents, progeny, and grandprogeny). The factors included: (i) population size; (ii) marker array density of the individual (3 genotyping statuses: not genotyped, genotyped at LD, or genotyped at HD); (iii) number of close relatives that were genotyped at each genotyping density (12 variables; 4 relationship levels and 3 genotyping statuses); (iv) number of close relatives that were sequenced and their cumulative sequencing coverage (8 variables; 2 variables for each of the 4 relationship levels); and (v) connectedness to the population, which was measured as the sum of coefficients of relationship between an individual and the rest of individuals in the pedigree.

We tested the predictive capacity of the partitioning factors for identifying individuals with low imputation accuracy, defined as those below 0.95. For that purpose, we performed a tenfold cross-validation on the simulated data. We validated the results of the regression trees in the real data against the imputation accuracy observed in the 284 high-coverage individuals. For the analysis of the variant-wise imputation accuracy we used only the individuals that were predicted to have imputation accuracy above 0.95 based on the partitioning factors of the regression tree. To further assess which factors affected the individual-wise imputation accuracy in the real data, we fitted a linear model predicting imputation accuracy against each of the factors used for the regression tree.

The factors in the regression tree for the variant-wise imputation accuracy included: (i) population size; (ii) MAF; (iii) relative position of the variant within a chromosome; (iv) distance of a variant to the nearest variant from the marker array (this distance was 0 if that variant was present on the marker array); (v) cumulative sequencing coverage across individuals at that variant site; and (vi) number of individuals with at least one sequencing read covering that variant site.

### Test 3: Impact of data misassignment and pedigree errors

We tested the impact that data misassignment and pedigree errors could have on the imputation results by introducing deliberate errors to the real data. We considered three types of errors: sequence data misassignment, marker array data misassignment, and pedigree errors. For each type of error, we created 284 scenarios, in which we altered the data of each of the individuals that were sequenced at high coverage in each population, one at a time. The three types of errors were defined as follows, to represent some worst-case scenarios:

#### Sequence data misassignment

We replaced the sequence data of the target individual by that of a random individual from the same population that had been sequenced at high coverage.

#### Marker array data misassignment

We replaced the marker array data of the target individual by that of a random individual from the same population that had been genotyped at HD, regardless of its own genotyping status or density.

#### Pedigree errors

We assigned random progeny from one of the individuals sequenced at high coverage from the same population to the target individual.

The impact of the data misassignment and pedigree errors on imputation accuracy was measured as the correlation between the allele dosages using the correct data and the erroneous data. The impact of these errors was assessed on the target individual where the error was introduced but also on its grandparents, parents, progeny, and grandprogeny to evaluate how the errors could propagate to relatives of the target individual. In the case of the pedigree errors, we also assessed the impact of the pedigree error on the misassigned progeny and grandprogeny. As a control, we also assessed the allele dosage correlation on the target individual and its relatives when the data of the target individual was removed, as done in Test 1.

## Results

### Individual-wise imputation accuracy in populations of different size

**Fig. 1:**
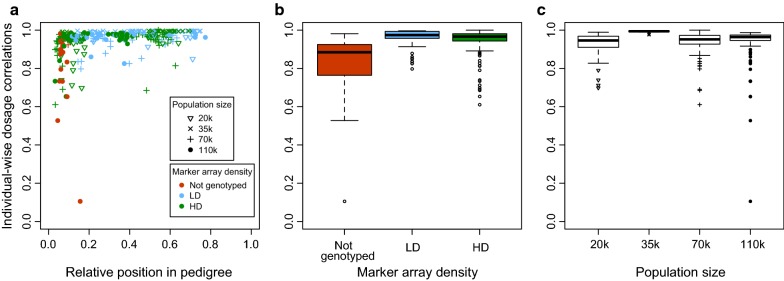
Individual-wise dosage correlation in real data with respect to **a** relative position of the tested individuals within a pedigree **b** genotyping marker array density, and **c** population size

The imputation accuracy in the real data was high for most of the tested individuals. The average individual-wise dosage correlation was 0.94 but there was substantial variation with an asymmetrical distribution (median: 0.97; min: 0.11; max: 1; interquartile range: 0.94–0.98). Many of the individuals in the earliest generations of the pedigree (some of the 106 individuals located in the first 20% of the pedigree) had a lower imputation accuracy than individuals in the remainder of pedigree. This pattern was observed for all four populations. Figure 1 shows the imputation accuracy plotted against relative position in the pedigree, the marker array density of the individual, or size of the population to which they belonged. The imputation accuracy of the individuals in later generations (the 178 individuals after the first 20% of the pedigree) was higher than that of individuals in the earliest generations, with an average dosage correlation of 0.97 and with much lower variability (median: 0.98; min: 0.69; max: 1; interquartile range: 0.96–0.99).

The marker array density of the individuals was confounded with the number of ancestors that were genotyped with marker arrays. The non-genotyped individuals (n = 19) and approximately half of the individuals genotyped at HD (n = 87 out of 157) belonged to early generations of the pedigree (Fig. 1a), which reduced their chances of having ancestors with genotype data and penalized the imputation accuracy for these two groups of individuals (Fig. 1b). On the contrary, most individuals genotyped at LD belonged to later generations (n = 91 out of 108), ensuring that there was enough data for their ancestors to enable high imputation accuracies for the LD individuals. The average imputation accuracy correlation was 0.81 for the non-genotyped individuals, 0.94 for the HD individuals, and 0.96 for the LD individuals. The average imputation accuracy for the HD individuals in the earliest generations was lower (0.91) than for the HD individuals in later generations (0.97). For individuals in the later generations, there were no significant differences between marker array densities and the average imputation accuracy of both the HD and LD individuals was 0.97.

There was no clear trend that population size affected imputation accuracy (Fig. 1c), especially for individuals in the later generations. The population with 35 k individuals had higher imputation accuracy than the other three populations but this was more likely due to population-specific characteristics, related to unbalanced distributions of the tested individuals across generations and genotyping statuses or potentially to pedigree structure, rather than population size. The 35 k population had only 5 out of 65 high-coverage individuals in the first 20% of the pedigree, compared to a much greater proportion in the other populations (from 15 out of 37 in the 15 k population to 56 out of 92 in the 65 k population).

### Factors that affect individual-wise imputation accuracy

The main factors that determined individual-wise imputation accuracy were whether the individual itself was genotyped with a marker array, the number of close relatives of that individual that were genotyped with a marker array (primarily parents and grandparents), and the connectedness of that individual to the rest of the population. The number of close relatives of an individual that were sequenced was a significant factor for the imputation accuracy of the 284 tested individuals in a linear model, but only the number of sequenced parents or progeny were influential partitioning factors in the regression trees based on the simulated data. The sequencing coverage of the relatives were not influential partitioning factors in the regression trees. The results were consistent between simulated and real data.

**Fig. 2:**
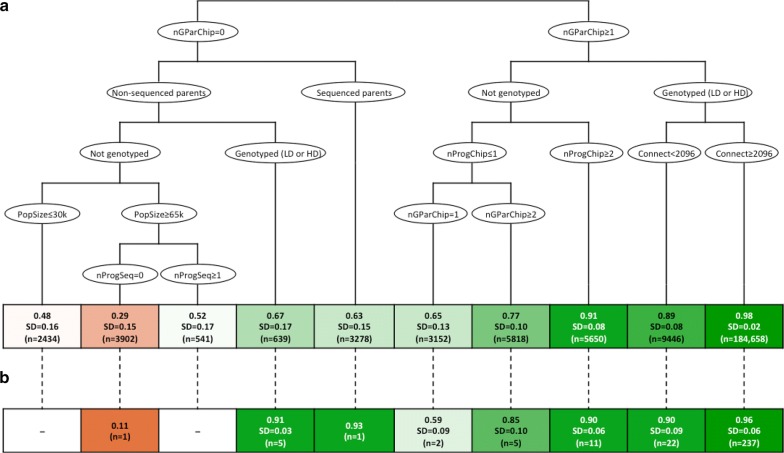
Regression tree of the factors that affected individual-wise dosage correlation in **a** simulated data and **b** comparison to real data. Variables include genotyping status, number of grandparents genotyped with marker array (nGParChip), number of progeny genotyped with marker array (nProgChip), number of sequenced progeny (nProgSeq), connectedness to the rest of the population (Connect), and population size (PopSize)

The regression tree for the factors that affect individual-wise imputation accuracy in the simulated data is shown in Fig. 2a. The first partitioning factor was the availability of marker array data of the grandparents. On average, individuals without genotyped grandparents had a much lower imputation accuracy (0.47, n = 10,794) than individuals with at least one genotyped grandparent (0.96, n = 208,724). For individuals without genotyped grandparents, other sources of information from the ancestors, such as availability of any sequenced parents, increased their imputation accuracy from 0.40 (n = 7516) to 0.63 (n = 3278).

After these initial partitions, the next partitioning factor was whether or not the individual itself was genotyped with a marker array, regardless of marker array density. For non-genotyped individuals, having some genotyped or sequenced progeny and grandprogeny improved their imputation accuracy. For genotyped individuals, regardless of the genotyping density, connectedness to the rest of the population was the main factor that determined imputation accuracy, with the dosage correlation increasing with connectedness from 0.89 (n = 9446) to 0.98 (n = 184,658). The imputation accuracy observed in the real data was consistent with the partitions of the regression tree based on the simulated data (Fig. 2b).

These partitioning factors predicted the simulated individuals with low imputation accuracy (lower than 0.95) with a sensitivity of 0.66 and a specificity of 0.95. In the real data, the correlation between the predicted and observed individual-wise imputation accuracy was 0.55 and the partitioning factors predicted the individuals with low imputation accuracy with a sensitivity of 0.42 and a specificity of 0.95. These partitioning factors were sensitive for predicting the individuals with the lowest imputation accuracy but their sensitivity decreased for predicting individuals with imputation accuracy closer to the desired level of 0.95 (Table 2). In total, 237 individuals were predicted to have imputation accuracy higher than 0.95 and were later used for the analyses of variant-wise imputation accuracy.

**Table 2:** Prediction of individuals with low imputation accuracy (below 0.95) using the partitioning factors from the regression tree

Data	Sensitivity	Specificity	Sensitivity by observed imputation accuracy				
			0–0.5	0.5–0.75	0.75–0.85	0.85–0.9	0.9–0.95
Simulated	0.66	0.97	1.00	0.99	0.94	0.68	0.27
Real	0.42	0.95	1.00	0.58	0.31	0.61	0.29

The analysis of the factors that affected the individual-wise imputation accuracy observed in the real data with a linear model largely supported the results of the regression trees. Table 3 summarises the factors that were significantly associated with individual-wise imputation accuracy. The significant factors included the number of genotyped ancestors (at HD; *p *≤ 0.016) but not the number of genotyped descendants (*p* = 0.062–0.996), and the number of sequenced relatives (*p* ≤ 0.016) but generally not their cumulative sequencing coverage (*p* = 0.044–0.456). The factors that referred to the amount of information available for the individuals themselves were also significant, including both their genotyping status (*p* ≤ 0.001) and their connectedness to the rest of the population (*p* = 0.031). However, the marker array density was confounded with the generation to which the individuals belonged and, therefore, with the number of ancestors that were genotyped with marker arrays (Fig. 1). Population size was also a significant factor (*p* ≤ 0.001), but likely confounded with population-specific factors (Fig. 1).

**Table 3:** Factors that affect individual-wise imputation accuracy on the real data

Factor	*p*-value
Population size	< 0.001
Individual data	
Genotyping status	< 0.001
Connectedness to the rest of population	0.031
Number of relatives genotyped with marker array	
Grandparents at LD	0.707
Grandparents at HD	0.016
Parents at LD	0.059
Parents at HD	< 0.001
Progeny at LD	0.062
Progeny at HD	0.553
Grandprogeny at LD	0.926
Grandprogeny at HD	0.996
Number of relatives sequenced	
Grandparents	0.003
Parents	< 0.001
Progeny	0.002
Grandprogeny	0.016
Cumulative sequencing coverage of relatives	
Grandparents	0.456
Parents	0.245
Progeny	0.100
Grandprogeny	0.044

*LD* low density, *HD* high density

### Variant-wise imputation accuracy

The variant-wise imputation accuracy was also high. After removing the individuals with a low predicted imputation accuracy, the average variant-wise dosage correlation was 0.91 (median: 0.98; min: − 0.37; max: 1; interquartile range: 0.94–0.99). We removed the individuals with a low predicted imputation accuracy to provide estimates of variant-wise imputation accuracy for the data that would pass such an initial quality control before any additional filtering steps or downstream analyses. Although removing such individuals resulted in slightly higher variant-wise imputation accuracy estimates than using all data (0.88 and 0.91 before and after filtering, respectively; correlation: 0.93), the results did not change.

**Fig. 3:**
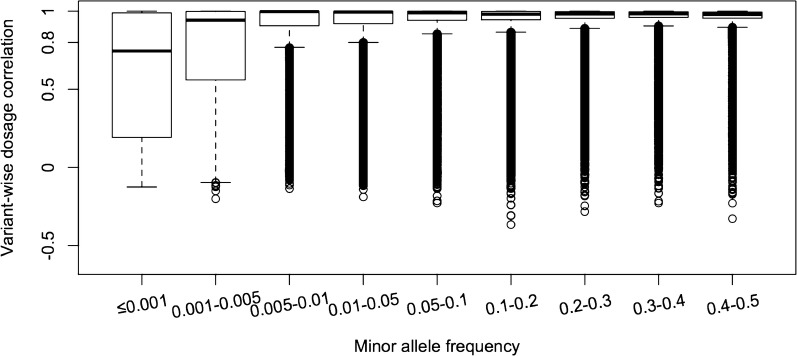
Variant-wise dosage correlation in real data with respect to minor allele frequency. Results are after removing individuals with low predicted imputation accuracy

Variant-wise imputation accuracy was lower for low-frequency variants, compared to more common variants. Figure 3 shows the distribution of the imputation accuracy for variants across the MAF spectrum. The mean imputation accuracy increased from 0.60 for MAF ≤ 0.001 to 0.95 for MAF ≥ 0.2, but the median imputation accuracy was > 0.98 for MAF ≥ 0.005.

### Factors that affect variant-wise imputation accuracy

The main factors that determined the variant-wise imputation accuracy were the MAF of the variants and the number of sequenced individuals at the variant site. Whether a marker was present in the marker array or not and the distance of a variant to the nearest variant from the marker array were not influential partitioning factors in the regression trees. The results were consistent between simulated and real data.

**Fig. 4:**
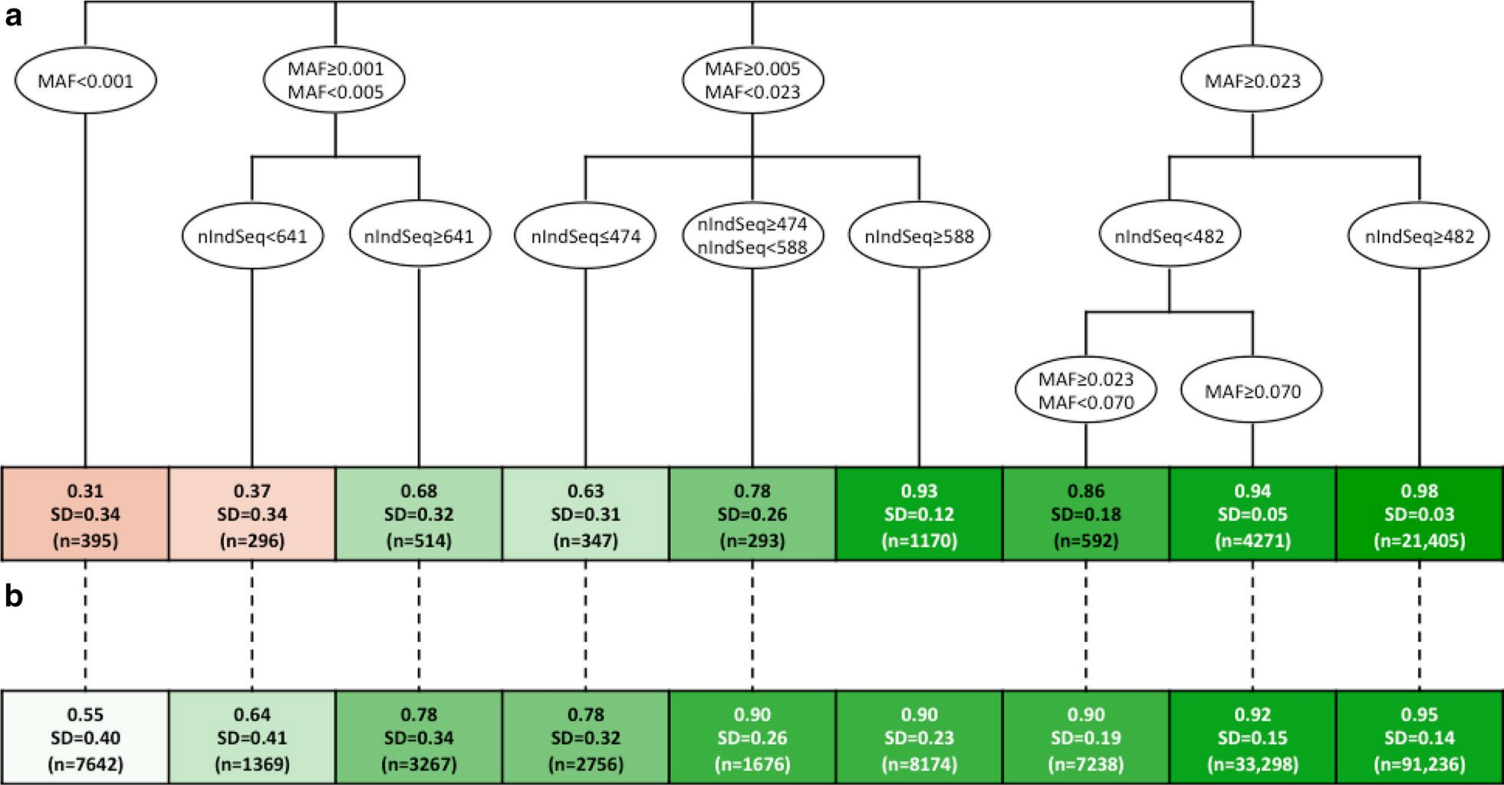
Regression tree of the factors that affected variant-wise dosage correlation in **a** simulated data and **b** comparison to real data. Variables include minor allele frequency (MAF) and number of individuals sequenced at a position (nIndSeq)

The regression tree for the factors that affect variant-wise imputation accuracy on the simulated data is shown in Fig. 4a. The first factor that determined variant-wise imputation accuracy was MAF. The imputation accuracy was limited for very rare variants: 0.31 for MAF below 0.001 (n = 395), 0.57 for MAF between 0.001 and 0.005 (n = 810), 0.85 for MAF between 0.005 and 0.023 (n = 1810), and 0.97 for MAF above 0.023 (n = 26,268). The other partition factor was the number of individuals that had at least one sequencing read that covered a given position. The imputation accuracy observed in the real data within each partition of the regression tree followed the same trends as for the simulated data, but ranged from 0.55 (n = 7642) to 0.95 (n = 91,236) and were greater than those from the simulated data at low MAF (Fig. 4b).

### Impact of data misassignment and pedigree errors

**Fig. 5:**
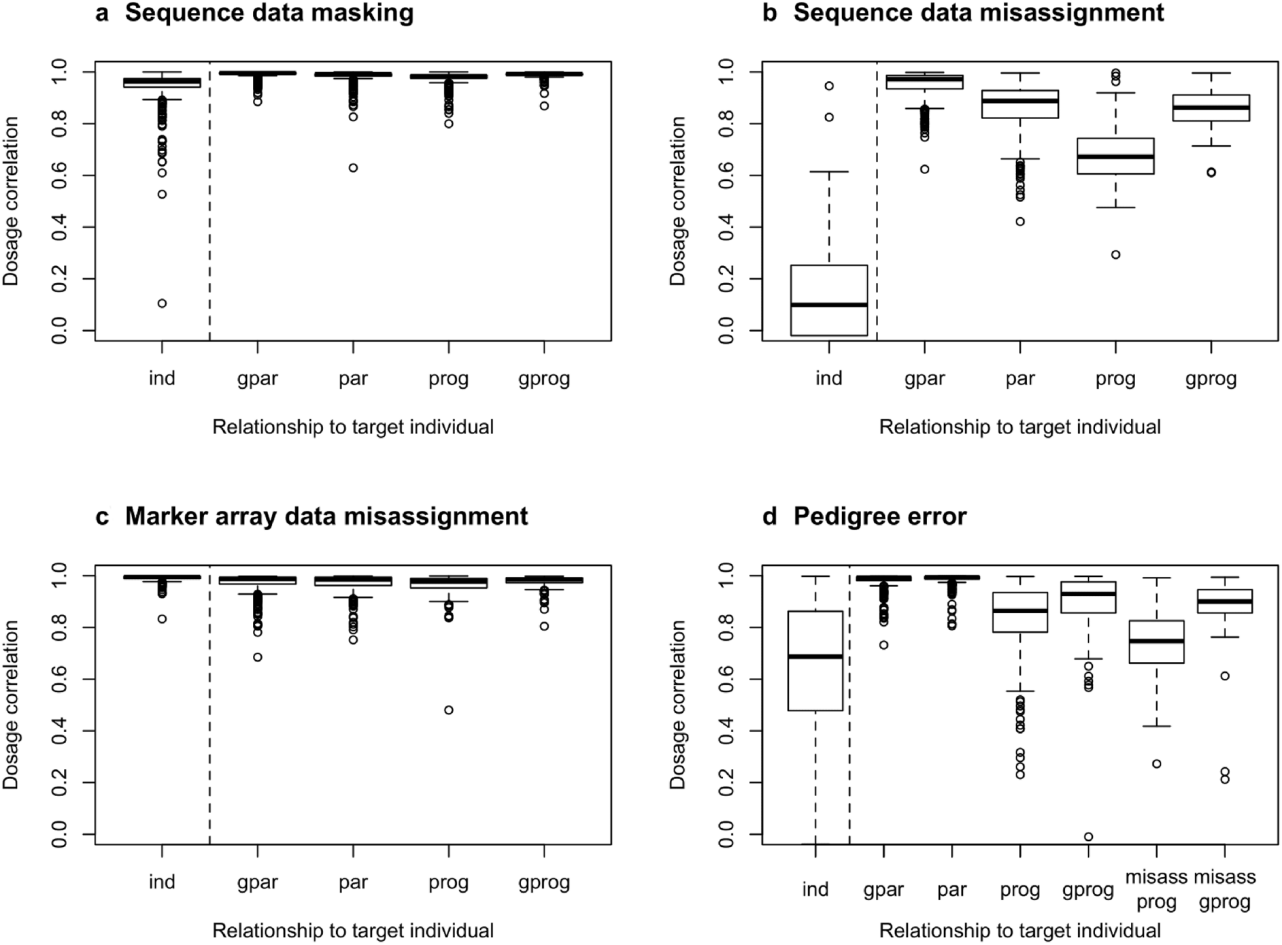
Impact of data misassignment and pedigree errors on imputation accuracy. The dashed line separates the individual directly affected by the data modification (ind) and its relatives (gpar: grandparents, par: parents, prog: progeny, gprog: grandprogeny, misass prog: misassigned progeny, misass gprog: misassigned grandprogeny). The y-axis measures the individual-wise dosage correlation between the imputed genotypes based on complete correct data and either missing or misassigned data for the individual itself and its relatives. In panel **a** we provide the case where the sequence data of the target individual was masked as in Test 1; in panel **b** where the sequence data of another individual was misassigned to the target one; in panel **c** where the marker array data was misassigned; and in panel **d** where we assigned the progeny from one of the individuals sequenced at high coverage to the target individual

Data misassignment and pedigree errors can have drastic consequences on the imputation results. The impact of data misassignment and pedigree errors, measured as the dosage correlation between the results with and without the deliberate error, is presented in Fig. 5 for the target individual (‘ind’) and its immediate relatives. We report here the average dosage correlation but note that there was large case-by-case variability due to the stochasticity of the data misassignment and pedigree errors.

When we removed the high-coverage sequence data of the target individual, as in Test 1 (Fig. 5a), the dosage correlation with complete data imputation was 0.94 for the target individual. The impact of removing the sequence data of the target individual had a limited impact on imputing its relatives, which had dosage correlations of 0.97 to 0.99 compared to the case with complete data.

When the sequence data was misassigned (Fig. 5b), the dosage correlation of the target individual drastically decreased to 0.13, as did (in order of magnitude) that of its progeny (0.68), then its grandprogeny (0.86) and parents (0.86), and finally its grandparents (0.95).

When the marker array data was misassigned (Fig. 5c), the dosage correlation of the target individual remained very high (0.99), probably because the high-coverage sequence data provided high certainty about its true genotypes. In spite of this, potential errors in the segregation probabilities resulted in dosage correlations for the relatives of the target individual that were slightly lower (0.97 to 0.98) and showed a greater dispersion.

Finally, when the pedigree was misassigned (Fig. 5d), the impact of such errors depended on the number of true and misassigned relatives that the target individual had. In our test, the target individual was misassigned progeny from one of the individuals sequenced at high coverage. The dosage correlation of the target individual greatly decreased (0.65). The greatest impact of the pedigree errors was on the misassigned progeny (0.74), but the impact on the true progeny was also large (0.83). The impact was smaller on the misassigned grandprogeny (0.89) and the true grandprogeny (0.90). The dosage correlation of the parents and grandparents of the target individual were largely unchanged (0.99 and 0.98, respectively), probably because they had other correctly assigned relatives (like their own parents) that contributed more accurate data.

## Discussion

In this paper, we present the results of a large-scale sequencing study that aimed at generating accurately imputed whole-genome sequence information on hundreds of thousands of individuals. Our results show that we were able to obtain highly accurate sequence information for approximately 230,000 individuals from four different populations that were genotyped at a maximum of 75,000 markers genome-wide, by sequencing only 2% of the individuals in each population, mostly at low coverage. We found that imputation accuracy was high for most individuals, especially for descendants of the first few generations of a pedigree. The same approach was applied to five additional populations (results not shown), providing high-quality whole-genome sequence data for more than 350,000 individuals. To our knowledge, this is the largest set of whole-genome sequence information assembled to date in pigs [33] or in any other livestock species (e.g., [7, 34]).

Our results give rise to five major points of discussion: (i) the overall performance of the sequencing strategy and the approach that we used for imputing whole-genome sequence data; (ii) the individual-wise imputation accuracy; (iii) the variant-wise imputation accuracy; (iv) the comparison to other imputation methods; and (v) the implications for population-wide sequencing studies.

### Overall performance of the sequencing strategy and hybrid peeling

The overall performance of our sequencing strategy coupled with hybrid peeling was high. We were able to impute whole-genome sequence data for hundreds of thousands of individuals with a median dosage correlation of 0.97 by sequencing only about 2% of the individuals in each of our pedigreed populations. Most of the sequenced individuals were sequenced at low coverage, with 90% of the sequenced individuals at either 1× or 2× and only 6.4% of the sequenced individuals being sequenced at a high coverage of 15× to 30×. Sequencing a subset of individuals at high coverage may improve the variant discovery rates as well as provide a validation set for variants discovered with low-coverage sequence data. It is difficult to separate the contributions of the sequencing strategy and of the imputation method to the imputation accuracy. We have assessed the contribution of the sequencing strategy on imputation accuracy in a companion paper [27]. Overall, sequencing coverage does not seem a very influential factor if a sufficiently large number of individuals is sequenced and, therefore, the sequencing strategy based primarily on low-coverage sequencing that we have described enabled high imputation accuracy in real livestock populations regardless of the size of the population.

Our sequencing strategy and imputation method enabled high imputation accuracy of whole-genome sequence data from marker arrays with relatively low densities, of approximately 15,000 and 75,000 markers genome-wide. The low dependence on marker arrays with higher densities contrasts with the findings of previous studies on imputation of whole-genome sequence data, which reported that marker array genotyping density was critical when using other sequencing strategies and imputation methods. For example, van Binsbergen et al. [35] found that imputing from marker arrays with a density similar to ours (50,000 markers genome-wide) resulted in low accuracies (dosage correlations of up to 0.80) when using the Beagle imputation software (version 3; [36]) in cattle. Van den Berg et al. [33] found similarly low accuracies in pigs (dosage correlations of ~ 0.70), probably because the number of sequenced individuals was small. In order to achieve higher imputation accuracies, an intermediate step of imputation to a much higher density (700,000 markers genome-wide or similar) was previously proposed [35]. This intermediate step has been used in several studies and with other imputation methods [33, 34, 37, 38], but this may be a drawback for populations where marker array data at such high densities are not available. We found that a combination of an appropriate sequencing strategy and hybrid peeling achieved high imputation accuracies without any intermediate imputation steps being required for the LD individuals. This was likely due to the ability of both methods to exploit pedigree and existing marker array information to maximise the value of the generated whole-genome sequence data for the whole population.

### Individual-wise imputation accuracy

Although most of the individuals had high imputation accuracy, a small portion of individuals had much lower imputation accuracies than the rest. These individuals mostly belonged to the earliest generations of each pedigree. This reduction of imputation accuracy in the earliest generations of the pedigree was consistent with observations in previous simulation studies [15, 27]. The individuals in these generations tend to have very little information available for themselves and for their ancestors, i.e., many of these individuals were not genotyped with marker arrays or their parents and grandparents were not genotyped either. Availability of marker array data from ancestors is critical for phasing and the accurate estimation of the segregation probabilities in the multi-locus step of hybrid peeling and it greatly affects the resulting accuracy of imputation accuracy.

In a similar way, the marker array density at which the ancestors were genotyped affected imputation accuracy of an individual, regardless of the marker array density at which the individual itself was genotyped. This can be explained by the fact that parental and grandparental genotypes are needed for accurately phasing the individual’s genotype and even a small number of markers is sufficient to capture the small number of recombinations between the individual and its parents [16]. Thus, strategies that target parents that contribute large numbers of progeny for genotyping at high density, such as current genotyping practices of breeding programs with genomic selection [39, 40], seem appropriate.

Provided that the segregation probabilities were accurately estimated, high connectedness of an individual to the rest of the population enhanced its imputation accuracy by favouring the transmission of information from many relatives and by increasing the likelihood that a closely connected individual has sequence data. In livestock breeding populations, it is usual that pedigrees are deep and individuals have a high degree of relatedness. The connectedness of the imputed individuals to a sufficient number of informative relatives with marker array or sequence data allows for high imputation accuracy (after the initial generations) even when only a small subset of individuals was sequenced at low levels of coverage.

It is possible to predict individuals with low imputation accuracy based on the availability of data for themselves and their relatives. This approach works especially well for filtering out those individuals with the lowest imputation accuracies as a first conservative data quality control step before downstream analyses. However, it is critical to perform quality controls of the data also before performing imputation to avoid any data misassignment or pedigree errors. In this study, we attempted to set an upper threshold for the impact that these errors could have on the individual-wise imputation accuracy of the affected individuals as well as how these errors propagate to the relatives of the affected individuals in a pedigree-based method. We found that the most serious errors occurred due to pedigree errors or assigning sequence data to a wrong individual. However, this may be distorted by the fact that all the target individuals had high-coverage sequence data. Therefore, misassignment of marker array data must not be ignored as it could also have a strong impact on imputation accuracy when it affects individuals that are not sequenced, sequenced at low coverage, or whose relatives are genotyped with low-density marker arrays. Fortunately, frameworks to detect data misassignment [41] and pedigree errors [42] have been developed and we have described an approach to correct such errors with little disruption of the pedigree structure. We did not test the impact that map errors could have on the imputation accuracy, but it is obvious that they would hamper the estimation of the segregation probabilities and thus imputation accuracy.

### Variant-wise imputation accuracy

We obtained high variant-wise imputation accuracy after filtering out individuals that were predicted to have low imputation accuracy. The primary factor for variant-wise imputation accuracy was MAF. This was expected, as MAF is widely known to be one of the main factors that determine imputation accuracy regardless of the imputation method, and we found, similar to other studies, that imputation accuracy was lower for variants with very low MAF [4, 35, 37, 43].

The next most important factor was the number of individuals that had sequence data at that variant site. Low-coverage sequencing results in a sparse distribution of reads along the genome, and it is likely that only a subset of the sequenced individuals will have any reads that map to a given variant site and that the cumulative coverage across variant sites will also vary. In our study, the number of individuals with some coverage and the cumulative coverage may be confounded because most individuals were sequenced at 1× or 2×, but in general this indicates the importance of having as many sequenced individuals as possible with some coverage at each variant site [27], a situation that is favoured by sequencing strategies based on low coverage.

The importance of the number of individuals that had sequence data at a variant site also suggests that imputation accuracy could be lower in regions with extreme base compositions or particular sequence motifs that hamper read alignment [44, 45]. While the complexity of a given region, namely the presence of large repeats, is another factor that could affect local imputation accuracy along a chromosome [37, 46], it was not considered in our study.

Inferring the segregation probabilities from the flanking markers that are included in the marker array did not result in noticeably lower imputation accuracy for those variants that were not included in the marker array. Moreover, variant-wise imputation accuracy was found to be independent of the distance between the variant and the flanking markers at which the segregation probabilities were estimated. These findings differed from those of previous studies using methods based on linkage disequilibrium (Beagle, version 3; [36]), where variant-wise imputation accuracy decreased as the distance between each variant and the nearest variant in the marker array (from which imputation to whole-genome sequence data was performed) increased [35].

### Comparison to other imputation methods

We did not intend to make a direct comparison of the performance of hybrid peeling with other available imputation methods because there are fundamental differences in how they exploit information (pedigree and linkage vs. linkage disequilibrium) and because sequencing strategies and imputation methods are confounded across studies. However, we have previously compared the performance of our hybrid peeling with findhap (version 4; [43]) [15] and other studies have compared other available imputation tools [37, 38, 43, 47], including tools such as Beagle (versions 3 and 4; [36, 48]), IMPUTE2 [49], findhap [43], FImpute [50], or Minimac3 [51]. Many of these methods are population-based imputation methods that use an already phased haplotype reference panel to impute genotyped individuals to whole-genome sequence data. As a consequence, previous studies of the factors that influence imputation accuracy have been primarily concerned with the design of the reference panel. Some of these concerns involve the convenience of using single-breed or multi-breed reference panels [38, 47], population-specific reference panels [38, 52], the availability of marker array data for the sequenced individuals or not (it removes the genotype uncertainty that otherwise would arise from sequencing at low coverage at some pre-established positions) [43], or the trade-off between number of individuals sequenced and sequencing coverage [43]. In contrast, in this paper, we used a purely pedigree-based imputation algorithm. This allows us to exploit the large amount of linkage between the haplotypes of an individual and their relatives.

### Implications for population-wide sequencing studies

The coupling of an appropriate sequencing strategy [13, 14, 27] and an appropriate imputation method, such as hybrid peeling [15], enabled the generation of large datasets of sequenced individuals at a low cost and with high accuracy. This is a critical step for the successful implementation of whole-genome sequence data for genomic prediction, within and across breeds, as well as for fine-mapping of causal variants underlying quantitative traits, which could guide the promotion and removal of alleles by gene editing [53, 54].

In this paper, we focused on individual-wise imputation accuracy as an indicator of the value of this data for applications such as genomic prediction. Previous studies on imputation accuracy of whole-genome sequence data focused on variant-wise imputation accuracy rather than individual-wise [35, 37, 43]. In the context of genomic prediction, the estimate of the realized relationship between two individuals will correlate strongly with the individual-wise, but not the variant-wise, imputation accuracy [31, 55]. Understanding which factors determine the variability of individual-wise, as well as variant-wise [35, 37], imputation accuracy would enable accuracy-aware filtering of the imputed data prior to downstream analyses. With that purpose, we used regression trees on simulated data designed to mimic the real data for identifying a small set of partitioning factors that may be used as predictors to filter out individuals with expected low imputation accuracy.

## Conclusions

We demonstrate the high accuracy of hybrid peeling for imputing whole-genome sequence data of hundreds of thousands of individuals from real livestock populations in which only a small fraction of the individuals (2%) had been sequenced, mostly at low coverage. Using data from pig populations, we show that imputation accuracy was very high for individuals that were genotyped with marker arrays with densities that ranged between 15,000 and 75,000 markers genome-wide. The coupling of an appropriate sequencing strategy and hybrid peeling is a powerful method for generating whole-genome sequence data in large pedigreed populations, as long as the individuals are connected to enough informative relatives with marker array or sequence data, and regardless of population size. The characterization of the factors that affect the individual-wise and variant-wise imputation accuracy of hybrid peeling can inform genotyping and sequencing strategies as well as provide accuracy-aware quality control guidelines for the imputed data before downstream analyses. The success of this sequencing strategy demonstrates the possibility of obtaining low-cost whole-genome sequence data on large pedigreed livestock populations, which is a critical step for the successful implementation of whole-genome sequence data for genomic prediction and fine-mapping of causal variants.

## Data Availability

The software packages AlphaSim, AlphaSeqOpt, AlphaPhase, AlphaImpute and AlphaPeel are available from the AlphaGenes website (http://www.alphagenes.roslin.ed.ac.uk). The datasets generated and analysed in this study are derived from the PIC breeding programme and not publicly available.
